# Ether Versus Chloroform

**Published:** 1875-02

**Authors:** E. W. Lee

**Affiliations:** M. D., (Dublin), Chicago


ARTICLE II.

### ETHER VERSUS CHLOROFORM.

A PAPER READ BEFORE THE COOK COUNTY MEDICAL SOCIETY,

BY E. W. LEE, M.D., (DUBLIN), CHICAGO.

I purpose this evening to draw your attention to the relative merits of Chloroform and Ether as anæsthetics. Of course my personal experience of the subject must be limited, but I have endeavored to collect such information as I thought would be interesting to the Society, which has been done in a rather hasty manner.

I suppose it will appear almost superfluous for me to say that this country is the birth-place of anæsthesia in surgery—ether having been discovered to have the power of destroying pain, by the late Dr. W. G. T. Morton, a dentist, of Boston, Mass., in 1846. Chloroform followed shortly after, Great Britain being the seat of the discovery, and to the late Sir James Young Simpson belongs the honor of having introduced it for a like purpose. These discoveries may be regarded as the greatest boon that has been conferred on suffering humanity. Although British writers on the subject give us credit for using ether comparatively largely in this country as an anæsthetic, I think the real facts would fail to sustain the assumption, chloroform being more generally employed, as it is considered more agreeable to the taste, more rapid in its action, and takes a smaller quantity to produce the desired effect. The main question to be considered this evening is the comparative safety of the agents. In order to arrive at a correct conclusion, it will be desirable to note the effect of each on circulation and respiration.

Owing to the frequent deaths occurring from the use of chloroform, a committee was appointed by the Royal Medical and Chirurgical Society of London, in 1864, to inquire into its uses, and physiological, therapeutical and toxical effects. This committee, while investigating the subject of chloroform, indirectly considered the use of ether. In their report we find as follows:

EFFECT OF ETHER INHALATION.

*The Heart.*—The muscular movement is but little influenced; the first, or stimulating effect, is less sudden and more sustained; even after insensibility is procured its action is more vigorous. Ether may be considered as a stimulant, in a certain degree, to the heart's action. The mercury of the hæmadynamometer at first is absolutely raised, never falling until the respirations cease.

EFFECT OF CHLOROFORM INHALATION.

*The Heart*—Is first stimulated, and its contractile force augmented, but after this its action is depressed, and although the respirations go on properly, its action, as shown by the mercury of the hæmadynamometer when connected with the circulation of the animal, fails, and the mercury falls.

*Respiration.*—In ether, with strong inhalation, there is a temporary arrest of respiration, but it is less marked than with chloroform. With small quantities there is no arrest of the breathing, although the number and depth of the respiratory efforts are diminished; after a short time the respirations become slow and full, and next, while their frequency rises, the range of their movements is reduced.

In chloroform, with strong inhalation, there is a temporary arrest of respiration, dependent on spasm. This arrest, after a few seconds, ceases, and inhalation can again take place. With smaller quantities the inspirations become gradually shallower, and for a time retain their natural order, but become less frequent, and after perfect insensibility is produced, the amount of air entering the chest is extremely small.

*How Ether Causes Death.*—The effects produced in a strong quantity equaled those of chloroform in a small, but with an important contrast, that it exerted but a very slight depressing influence on the heart. Death occurred by the failure of the respiratory movements, the heart's pulsations continuing generally for some time after the respiration had ceased.

With chloroform, strong inhalation caused the pulse and respiration to cease nearly simultaneously. In the majority of cases the pulse stopped before the respiration, and the heart's action could be distinguished for some time after the pulse had ceased.

The conclusion formed by the committee was that it is desirable to obtain an agent which shall produce the required insensibility, and yet be not so dangerous in its operation as chloroform. Ether, to a certain extent, fulfils these conditions. It is less dangerous than chloroform, but its odor is disagreeable; it is slow in operation, and it gives rise to greater excitement than chloroform.

Other experiments have been made, showing that an atmosphere charged with more than a certain percentage of the vapor of chloroform, stops the heart before breathing ceases. The chloroform committee reported that the sequence of the phenomena during the experiments on animals is similar to that observed in man; and if the same percentage of the agent be administered, the results induced are nearly uniform. By combining the statistics collected by Dr. Andrews, of this city, and Dr. Richardson, of London, we find that in 92,815 ether inhalations, there were 4 deaths, or 1 to 23,204; in chloroform, 53 deaths to 152,260, or 1 to 2,873; a mixture of chloroform and ether, 2 deaths to 11,176, or 1 to 5,588.

In the report of the committee, June, 1864, a table of 123 deaths, occurring during or immediately after the administration of chloroform, is recorded, 109 deaths being thus given: Under 5 years, none; from 5 to 10 years, 9; from 15 to 30 years, 30; from 30 to 45 years, 32; from 45 to 60 years, 12; over 60 years, 2; 72 were males, 32 females.

The statistical tables of Drs. Andrews and Richardson demonstrate pretty clearly the relative safety of the agents. They show chloroform to be about eight times as dangerous as ether, and about twice as dangerous as chloroform and ether.

Many surgeons have abandoned the use of chloroform altogether as an anæsthetic. Prof. Gunn, of Rush Medical College, and Prof. Andrews, of Chicago Medical College, have, I believe, used ether exclusively for years, without accident, if I am correctly informed.

It has been proved that ether is superior to chloroform in sustaining the heart's action; therefore it has the less power for evil in case of careless or unskillful administration. The various stages of its operation are so distinctly marked that if the most ordinary care be used in its employment, it is next to an impossibility for fatal results to ensue.

According to Dr. Ashurst and Dr. Morgan, in administering ether you can exclude air with perfect safety; with chloroform the most extreme care must be used to admit air. Dr. Morgan, of Dublin, a surgeon and teacher of many years' experience, has devoted considerable attention to this subject. He has devised an ether inhaler which appears to answer the purpose so well as to effectually dispose of the objections attending ether as an anæsthetic agent. He has written a pamphlet containing much valuable information, and from it I have obtained much of the matter which I am able to lay before you this evening. Dr. M. says, regarding the admission or non-admission of air in employing ether, there has been much diversity of opinion, and some of the objections which have been urged against its use, of causing delay, sickness of stomach, headache or spasm, are dependent on this very diversity of application, as it is found by those surgeons who exclude air, that the results are satisfactory in the extreme. We have here the key of the question: ether vapor being considerably heavier than air, when applied by the sponge would flow away unseen from the mouth and nose, and much of it thus escape inhalation, while from being concentrated and more efficiently applied by the other mode (exclusion of air) it would act more satisfactorily, and produce anæsthesia.

I had a case the other day which tested this very point severely. A stout healthy man came into my office; the last joint of one of the index fingers had been crushed whilst coupling cars; on examination, I found it would be necessary to amputate above the seat of injury; he had just eaten a hearty dinner; he would not submit to the operation without an anæsthetic, so I put him under the influence of ether, fully expecting that in doing so he would deposit his dinner, if not my fee, before leaving.

Carefully excluding air, using this inhaler, he was insensible in three and one-half minutes. The operation was performed, when he quickly recovered consciousness; there was no sickness of stomach whatever. Dr. Morgan asserts that ether when applied in this manner produces insensibility as quickly as chloroform, and from what experience I have had with it I certainly agree with him. He considers it established, that while chloroform exerts a depressing influence on the heart, ether exerts a stimulating one; that chloroform is the most dangerous, while ether is the safest of all anæsthetics; that the only argument in favor of chloroform as contrasted with ether is, its convenience. Mr. Erichsen says in his work, "the fatal consequences which have attended the use of chloroform have caused American surgeons to almost entirely trust to ether in preference. Ether is certainly the safer agent." He also says, the only argument in favor of chloroform, is its convenience.

A French surgeon, Mons. Diday, of Lyons, has gone so far as to say, that any surgeon using chloroform instead of ether as an anæsthetic, would be culpable. A commission of inquiry formed by the Medical Society of that city, pronounced in favor of etherization, admitting that the comparative study of the two anæsthetics was far from being complete.

Notwithstanding the results of experiments and experience, many of the most eminent surgeons record their preference for chloroform as an anæsthetic agent. Mr. Lister, in a very able article in the last edition of Holmes' Surgery, takes strong ground in that direction, contending that the only cases where death results, unavoidably, from the use of chloroform is, when an idiosyncracy exists antagonistic to its use, and that so seldom happens that it is not worthy of consideration at all. He states, in fact, that death only occurs from careless or unskillful administration, and he ruthlessly upsets many of our time-honored rules for conducting its use. Our preliminary examination of the heart, for instance, might better be dispensed with altogether, for according to him, a patient with a diseased heart in the event of having to undergo an operation needs the chloroform more than if such a condition did not exist, the risk of the shock of the operation on the feeble heart being greater than that of the anæsthetic ; better let the pulse alone and let the administrator concentrate his entire attention on observing the respiration, as that is the first to go wrong, the pulse failing secondarily—the heaving of the chest goes on when actually no air is entering. This would appear to coincide with results observed by the chloroform committee. He lays great stress on the beneficial effect of drawing out the tongue forcibly in case of accident, contending that in many cases it fails from not being properly done. Notwithstanding his able arguments in favor of chloroform, a careful perusal of his article and the cases which he cites, where death was imminent but for prompt and decided action, makes one more distrustful of its safety. Mr. Syme, he says, used chloroform in many thousand cases, also the late Sir J. Y. Simpson, without any unpleasant consequences. Simpson lost one case, near the end of his career.

Mr. Lister disposes of ether in a very summary manner, but he is obliged to admit nearly all the points claimed for it. While I do not agree with his conclusions, for argument's sake, admitting that they are correct, the very elaborate directions he finds it necessary to give for its administration, and the narrow escapes he has witnessed, must be regarded as a strong argument in favor of some agent possessing less dangerous qualities. I cannot refrain from relating a case bearing strongly on this point. Some ten years since, a young friend of mine was desirous of having an operation performed for strabismus, which was of an aggravated character, causing him much annoyance and discomfort. One of the first oculists of the day was engaged to operate, and he brought along an able assistant to take charge of the anæsthetic. Chloroform was chosen, but on attempting to administer it, the heart's action became so tumultuous that after repeated efforts to get him under its influence they were obliged to desist, and the operation was indefinitely postponed. During the past summer I saw him for the first time since; he was then suffering from double strabismus, the other eye having become affected, but not in so marked a degree. I urged him to try once more, and that ether should be employed instead of chloroform; after some persuasion he yielded, but insisted that he could not be anæsthetized. Yet, notwithstanding these unfavorable circumstances he sank into insensibility in about four minutes, and the operation was successfully performed, resulting in perfect correction of the deformity. Here was a case in which there certainly existed an idiosyncrasy antagonistic to chloroform yet yielding readily to ether.

To the general practitioner this subject must be full of interest: he is depending in many instances on his local reputation for his success. The operating surgeon, having a firm standing in the estimation of the public, can stand a fatal case or two with far less injury to his reputation than his less prominent brother, therefore ought he not to select with great care the safest agent, not only for the sake of those whom he is called on to aid (which, of course, should be the first consideration,) but also for his own?

It may not be out of place here to make some remarks concerning the use of anæsthetics in cases of severe shock. Either of the agents in question has the power of stimulating the circulation when properly used, and is relied on as a most potent aid in procuring that result. So the surgeon does not now wait, as of old, for reaction, but proceeds to use the anæsthetic, some contending that we cannot use it too soon. This I cannot agree with, as nature, in most cases, makes some effort at reaction, and when she does, is the proper time to employ it. You certainly run a risk of annihilating the action of the already feeble heart by too early administration, and the fact of its having been used under such circumstances with impunity should not be considered any justification of the practice. There is hardly a practitioner who has had occasion to use chloroform frequently, that cannot call to mind some very narrow escapes. As has been shown, the space between complete insensibility from chloroform, and death, is short indeed. Sometimes we are called on under circumstances where we are obliged not only to operate, but look after the anæsthetic as well, skilled help not being available, so that the responsibility is double. I think few will deny that, under such circumstances ether is immeasurably the safer agent.

I am, as I said in the beginning of this paper, contrasting the relative merits of the two leading anæsthetics. I think that the statistics relative to the ages at which death occurred, to which I have referred, need some attention. They appear to show that the period under five years of age may be regarded as safe for chloroform administration. This may, perhaps, be explained by the fact that fewer subjects are submitted to its influence at that age. I have never heard of any accident attending its use during parturition, there seeming to be a peculiar condition favorable to its action.

If I would not be considered tedious, I should like to relate a case bearing on that point. I attended a healthy young woman in parturition, some years since; the complications arising during the labor, necessitated the use of chloroform at intervals, for about five hours. She bore it remarkably well. I had no professional assistance. That same woman came to my office, some time after, to have a needle extracted, that penetrated in the vicinity of the knee-joint. I had an assistant to administer chloroform to her. She was barely under its influence, when, without any warning, circulation and respiration suddenly ceased, and were with great difficulty again restored.

Considering the absence of any record of deaths during parturition, and under the period of five years of age, chloroform, in these cases, may be considered as safe as any other anæsthetic; under all other circumstances, ether is superior.

Before closing, I will draw the attention of the Society to the fact that ether is, and has been for a long time, in certain districts in the north of Ireland, used as an intoxicant. Its exhilarating effects are such, Dr. Morgan says, that many have found it a seductive substitute for alcoholic stimulants, even to the extent of producing intoxication. In Ireland, curiously, its virtues, if they may be styled in this way, have been already appreciated, and as that province whose inhabitants are peculiarly accredited with astuteness has been the most noted for its consumption, we may conclude that it presents, despite its supposed disagreeable odor, the recommendation of being good value in producing the desired effects.

A Mr. Draper, in the *Medical Press and Circular*, has lately put the question of ether as an intoxicant in so clear a light, that it has attracted considerable attention. He states: "The floating idea that there are fair consumers of Hoffman's Anodyne aud perles d'Ether, for whom ether had never been prescribed, quite prepares one for the discovery that there are, in the northern part of Ireland, a number of people who, forswearing alcohol, supply its place with ether—a race to whom ether is what koumiss is to a Kalmuck, ava to a South Sea Islander, absinthe to a certain class of Frenchmen, or gin and whisky to their more immediate neighbors. That they should take 'nips' of ether morning, noon and night, as they would whisky, and—for anything shown to the contrary—drink good luck or ratify bargains in a glass of ether, was not a thing to look for, and is perhaps without parallel in the history of narcotic stimulants. The facts rest upon the authority of a number of gentlemen in their respective capacities of physicians, clergymen, ether manufacturers and druggists.

The usual quantity of ether taken at one time is from two to four drams, and this dose is repeated twice, thrice, or even four and six times daily. It is taken unmixed with water; indeed, its very slight solubility in that fluid would make this a useless precaution; but the usual practice is to first take a mouthful of water, then a dose of ether, and again a mouthful of water.

The intoxication produced by ether resembles that of alcohol, but it is much more rapidly produced, and is more evanescent. The ether seems to be eliminated entirely by the lungs, and the breath of the ether drinker always affords ample evidence of his addiction to the habit. I am credibly informed that at the fair of Drapertown, which appears to be the paradise of ether drinkers, the prevalent smell is not, as at country fairs, of pigs, tobacco smoke, or of unwashed human beings, but of ether. I have not been able to learn that, apart from the moral ill effects common to all excitants and intoxicants, the habitual use of ether brings in its train any peculiar evils, and although it would be wrong to draw a conclusion from completely negative evidence, I am disposed to believe that the votaries of ether incur less danger from the habit than ordinary dram drinkers, and there are two good reasons for this belief.

If we assume that there is nothing specificially injurious in the action of ether, it will be readily admitted that, having a definite chemical composition, and not being very liable to adulteration with other fluids, it must be an improvement upon the sophisticated alcoholic potations which, with these people, it has replaced. Again, the affinity of ether for water is so slight that dehydration of the mucous tissue of the alimentary canal and that aseptic condition which so well mark the difference between the effect of ardent spirits and of alcohol in the form of unbrandied wine, cannot be evils attending its use.

*The quantity consumed.*—Now if we assume the ordinary quantity taken at one time to average three drams, and this quantity to be in effect the equivalent of half a glass of whisky, we arrive at the result that three gallons of ether supply the place of ten gallons of whisky. It is very difficult to arrive at any accurate idea of the extent to which ether is used in the north of Ireland. Omagh is said to take 250 gallons yearly, and one Dublin manufacturer has sent to Belfast at the rate of 4,000 gallons yearly."

Dr. Lee then exhibited the inhaler, and stated that he had used it in some twenty cases. The shortest time, in adults, for anæsthetization was two and one-half minutes; the longest, eight. In children the time was even shorter. It is so constructed that air can be entirely excluded; about one ounce of ether usually produces complete anæsthesia, and if the operation be prolonged, one-half ounce is poured into the apparatus as required. Squibb's ether was used.

